# Dietary pattern transition and its nutrient intakes and diet quality among Japanese population: results from the 2003–2019 National Survey

**DOI:** 10.1017/S1368980024002027

**Published:** 2024-10-21

**Authors:** Yui Sakai, Yen Yen Sally Rahayu, Yajie Zhao, Tetsuya Araki

**Affiliations:** 1 Department of Global Agricultural Sciences, Graduate School of Agricultural and Life Science, The University of Tokyo, Bunkyo Ward, Tokyo, Japan; 2 Tokyo College, The University of Tokyo, Bunkyo Ward, Tokyo, Japan

**Keywords:** Dietary patterns, National survey, Traditional, Japanese diet, Nutrition assessment, Westernisation of diet

## Abstract

**Objective:**

While many Asian countries undergo dietary transitions, little is known about Japan’s dietary pattern changes and their impact on nutritional intake. We aimed to examine 17-year trends in dietary patterns and nutrient intakes in Japan.

**Design:**

Principal component analysis was used to derive dietary patterns. The nutrient intake of subjects with principal component scores in the highest quartiles of each dietary pattern was assessed using the NRF9·3, which is a scale that evaluates the entire diet in terms of nutrient density.

**Setting:**

Japan.

**Participants:**

We used data from the National Survey 2003–2019 (67 066 women, 55 133 men).

**Results:**

Two common dietary patterns were identified in both sexes: the ‘Japanese style’ and the ‘bread and dairy’ pattern. Additionally, two other patterns: ‘meat and oil’ and ‘noodles’, were pronounced in men. Over the 17 years, the scores of the ‘Japanese style’ pattern decreased while the ‘meat and oil’ and the ‘bread and dairy’ patterns increased. The nutrition assessment result showed that the highest quartiles of the ‘Japanese style’ pattern had higher NRF scores (women: 716·0, men: 670·5) (*P* < 0·001), whereas those of the ‘bread and dairy’ pattern had a lower score (636·9 in women, 661·2 in men) (*P* < 0·001).

**Conclusions:**

Trend analysis in this study suggests the ongoing Westernisation of diet in Japan. A decreasing trend of the dietary pattern with the most preferable nutrient profile (i.e. the ‘Japanese style’) might indicate a potential decrease in beneficial nutrient intake and, thus, a deterioration of the nutritional status of the Japanese population.

Dietary transition refers to the shift in a country’s diet due to economic and social changes^(1)^. This shift is characterised by a transition from traditional plant-based diets to diets that include more animal products, fats, sugars and processed foods^(2,3)^, reflecting a Western dietary pattern^(1)^. This global trend has negative impacts on human health and the environment, including obesity and food waste^(4–6)^.

In Asia, the dietary transition began with income-driven diversification, followed by the adoption of non-traditional dietary patterns due to globalisation and the Westernisation of diets^(7,8)^. In Japan, the typical diet in the 1960s and 1970s included high intakes of vegetables and seafood but low animal foods, which was associated with a low prevalence of coronary artery disease^(9)^. However, the per-capita supply of meat/poultry and fats/oils increased while the rice supply decreased, indicating the ongoing Westernisation of the Japanese diet^(2)^. The continuous Westernisation of the Japanese diet was also reported in the latest National Health and Nutrition Survey, Japan (NHNSJ) study (2003–2015)^(10)^ and has been inversely associated with health indicators such as total cholesterol and LDL-cholesterol^(11)^.

Recently, nutritional epidemiology has focused on the assessment of dietary patterns to examine the potential synergistic effects of foods and nutrients^(10)^. Although the Japanese dietary pattern transitions have been identified^(10)^, overall dietary quality has yet to be assessed using long-term data^(11,12)^. To fill this gap, we used the most recent nationally representative dataset (2003–2019) to examine if the Westernisation of the Japanese diet continues and evaluated nutrient intakes to assess overall diet quality. As one of the Asian countries with a pronounced dietary transition^(1)^, understanding these trends is crucial for developing countermeasures to improve diets and reduce diet-related diseases. Japan’s experience can be applied in other Asian regions, where the transition may be accelerated in the future.

## Materials and methods

### Data source and study design

Data from the NHNSJ from 2003–2019 were used for the study. The design and procedure details of the NHNSJ were described in elsewhere^(13)^. In brief, the survey was conducted annually, mainly in November, by local public health centres representing all regions of Japan. With an estimated 50 % response rate, 3919–12 750 households participated in the study. In total, 122 199 people (67 066 women, 55 133 men) were included in the analysis after applying the exclusion criteria of being under 18 years old, pregnant, or breast-feeding and missing responses.

### Anthropometric measurement

Body height and body weight were measured on most of the participants by weight and height scales by trained ﬁeldworkers using standardised procedures^(14)^. In brief, height was measured by having the subject stand upright with socks off, both heels close together, and the back, buttocks and heels in contact with the scale post of the stadiometer. Weight was measured by placing the subject in a near-naked position and having him or her stand quietly at the centre of the scale’s weighing platform. For other participants, height and weight were measured either by other household members at home or self-reported. Height (to the nearest 0·1 cm) and weight (to the nearest 0·1 kg) were measured while the participants were barefoot and wearing light clothes only, and BMI was calculated as weight (kg) divided by height squared (m^2^).

### Dietary assessment

Dietary intake data were obtained using a one-day, semi-weighed household dietary record. Trained fieldworkers instructed participants to record all food consumed for a typical day, excluding special occasions. The main household record keeper (usually the member responsible for meal preparation) was instructed to weigh all food and beverages consumed by household members by food scale and to record names and weights on open-ended form, including the amount of food waste and leftovers. Trained field researchers visited each household, checked the recording forms for completeness, verified portions using commercial food models or food booklets if necessary and corrected missing or illogical information. In accordance with the NHNSJ manual, trained researchers estimated the recorded food portions or quantities to food weights and coded each food item according to the NHNSJ food number list to calculate energy and nutrient intakes from household food consumption records. These food number lists are based on the newest Standard Tables of Food Composition in Japan (STFC) at the date (5^th^ edition^(15)^ for 2003, 2004, 5^th^ revised and enlarged edition^(16)^ for 2005–2010, 6^th^ edition^(17)^ for 2011–2015 and 7^th^ edition for 2016–2019^(18)^). Although the trends in energy and nutrient intakes observed in this study were generally consistent, the possibility of data continuity issues cannot be excluded because the NHNSJ survey results were not recalculated based on the updated STFC. When analysing NHNSJ data from multiple years, continuity issues may arise due to revisions of the STFC, which could change the survey results^(19,20)^. Although the continuity of calculations using different versions of the STFC in Japan has not been well studied^(21)^, we are against recalculating past dietary survey data using the most recent STFC, given the lack of abrupt changes in dietary intake trends. For example, slight changes (less than about 2 %) in average intakes of energy, protein and fat have been reported in the revision from the 4^th^ to the 5^th^ edition^(19,21)^.

### Identification of dietary patterns and nutrition values

A principal component analysis was performed to derive dietary patterns. Eigenvalues greater than 1·5 were utilised to assess whether a component should be considered a major dietary pattern^(11)^. Varimax rotation was applied to review the correlations between variables and factors. Food items were categorised into thirty food groups based on functional food ingredients (see online supplementary material, Supplemental Table S1), and correlation coefficients were calculated between food groups and dietary patterns. Food groups with absolute principal component loadings higher than 0·3 were determined to contribute to the component^(10)^. Dietary pattern scores (principal component scores) were averaged for each survey year. Data were graphed by year for women and men to examine trends in dietary pattern scores over the study period. In both models, dietary pattern scores were standardised by converting to a mean of 0 and variance of 1, with a negative indicating low adherence to the dietary pattern and a positive indicating high adherence. Principal component scores were then compared for the highest quartile group based on the highest quartile of principal component scores for each gender, and average nutrient intakes between dietary patterns were compared. The NRF9·3 (Nutrient-Rich Food Index 9·3) scores were calculated for each individual to estimate the average score for each dietary pattern, with higher scores indicating better overall diet quality, as detailed in Murakami *et al.* (2020)^(22)^. The score is based on nine beneficial nutrients (i.e. protein, fibre, vitamin A, vitamin C, vitamin E, Ca, Mg, Fe and K) and three limiting nutrients (i.e. saturated fat, added sugars and Na), with a score added or subtracted for every beneficial and limiting nutrient intake, respectively. Due to limited data on added sugar intake, total sugar intake was used in the calculation.

### Statistical analysis

We used ANOVA for categorical variables and linear regression for continuous data to analyse trends in participant characteristics and dietary pattern scores over time. Tukey–Kramer tests were performed for nutrient intake in the dietary pattern, with a significance level of *P* < 0·001. Python version 3·9·0 was used for statistical analysis.

## Results

### Participants characteristics

The mean BMI and age of participants demonstrated slightly increasing trends (*P* < 0·001). Similarly, the mean age increased over the survey years, and participants’ ages were categorised as 18–39 years, 40–59 years and ≥60 years. The characteristics of participants in this study are summarised in see online supplementary material, Supplemental Table S2.

### Principal component analysis of dietary patterns

The principal component analysis identified two dietary patterns in women and four in men, explaining 12·2% and 25·2 % of the total variance, respectively. Table 1 shows food group loadings, equivalent to correlations between the food group and dietary patterns. The first component (PC1) was characterised by a high intake of vegetables, fruits, pulses, tubers and roots, mushrooms, seaweeds, sea products, Japanese seasonings and sugar and in men rice and pickled vegetables. PC1 was named the ‘Japanese style’ pattern. The second component (PC2) was dominated by bread, fruits and dairy products and named the ‘bread and dairy’ pattern. In men, two other patterns were derived: the ‘meat and oil’ pattern (PC3), loaded positively on meat products and vegetable oil and the ‘noodles’ pattern (PC4), loaded positively on noodles and salt-based seasoning.

**Table 1:** Principal component loadings for each of the thirty food groups for dietary patterns identified among the 67 066 women and 55 133 men adults in the national health and nutrition survey, 2003–2019

	Women		Men			
Food groups	Japanese style pattern	Bread and dairy pattern	Japanese style pattern	Bread and dairy pattern	Meat and oil pattern	Noodles pattern
Rice	0·27	–0·58	**0·36**	–0·36	0·15	–0·50
Bread	–0·14	**0·55**	–0·33	**0·58**	0·12	–0·03
Noodles	–0·16	0·23	–0·10	–0·04	–0·07	**0·79**
Other grains	0·04	0·22	–0·01	0·04	**0·32**	0·08
Tubers and roots	**0·38**	0·00	**0·34**	0·09	0·19	–0·13
Sugar	**0·40**	0·23	0·25	**0·43**	0·05	–0·07
Pulses	**0·39**	–0·09	**0·44**	0·02	–0·08	0·02
Nuts	0·20	0·13	0·11	0·21	–0·03	0·02
Vegetables	**0·61**	0·14	**0·57**	0·18	0·28	0·08
Pickled vegetables	0·27	–0·18	**0·31**	–0·01	–0·14	–0·04
Fruit	**0·46**	0·15	**0·31**	**0·53**	–0·17	0·00
Mushrooms	**0·32**	0·09	**0·36**	0·07	0·07	0·20
Seaweeds	**0·32**	–0·06	**0·31**	0·04	–0·04	–0·02
Sea products	**0·42**	–0·20	**0·44**	0·01	–0·21	–0·08
Beef	0·01	0·11	–0·01	0·00	0·18	–0·05
Pork	0·04	0·14	0·04	–0·08	**0·37**	0·13
Processed meat	–0·04	**0·31**	–0·10	0·05	**0·40**	0·10
Chicken	0·01	0·09	–0·02	–0·11	**0·32**	–0·07
Eggs	0·14	0·16	0·14	0·00	**0·45**	–0·07
Dairy products	0·20	**0·39**	–0·02	**0·59**	0·01	0·02
Animal fat	–0·03	**0·38**	–0·14	0·23	0·26	0·07
Vegetable oil	0·09	**0·41**	–0·04	0·06	**0·68**	–0·06
Confectioneries	0·05	0·17	–0·02	0·30	0·00	0·00
Alcoholic drinks	–0·07	0·08	0·09	–0·22	0·04	0·23
Tea and coffee	0·29	0·11	0·18	0·29	–0·04	–0·12
Soft drinks	–0·10	0·15	–0·12	–0·05	0·27	–0·02
Salt-based seasonings	0·12	0·18	0·13	–0·07	0·09	**0·70**
Japanese seasonings	**0·63**	–0·24	**0·69**	–0·08	–0·02	0·08
Eigenvalue	2·28	1·71	2·21	1·70	1·60	1·54
Variance (%)	8·14	6·10	7·90	6·07	5·71	5·49
Total variance (%)	8·14	14·24	7·90	13·97	19·68	25·17

Loading values >0.3 are presented in bold. Total explained variance of dietary patterns was 12.24 % in women and 25.17 % in men.

### Dietary patterns trends

**Fig. 1:**
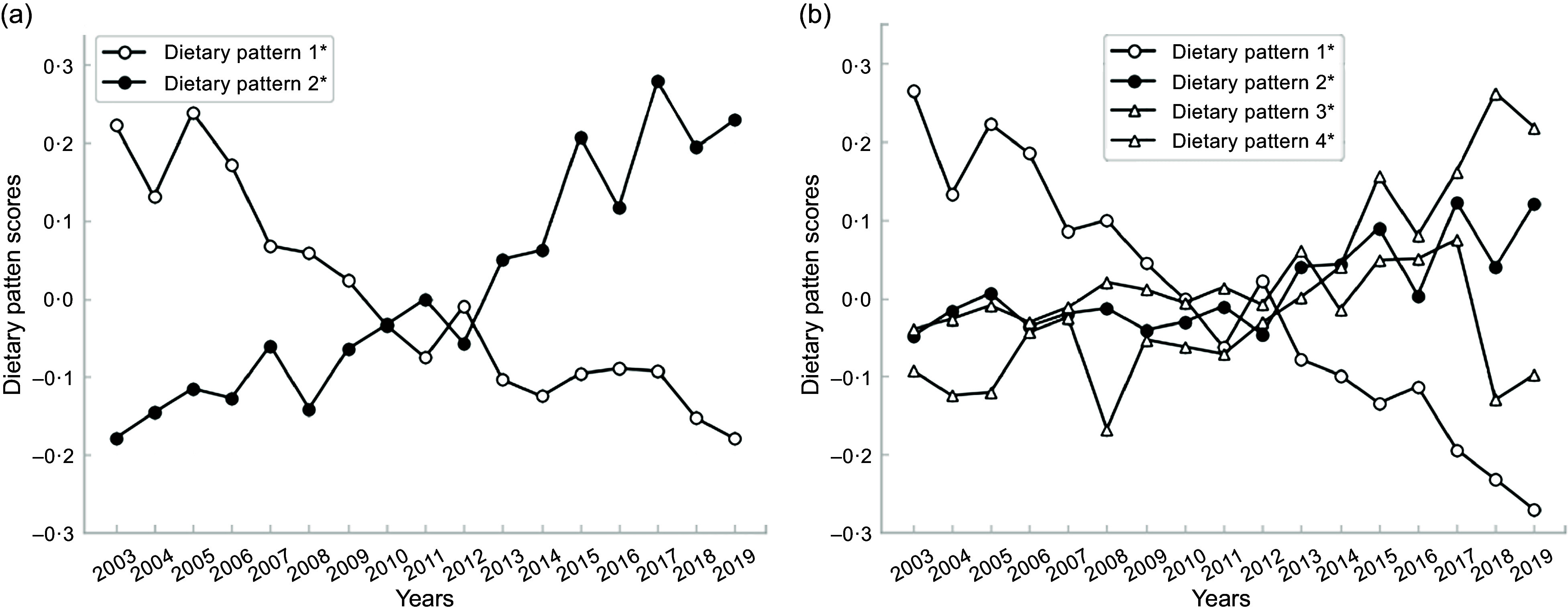
The trends from 2003 to 2019 in dietary pattern scores in women (*n* 67 066) (a) and men (*n* 55 133) and (b), a cross-sectional study, National Health and Nutrition Survey, Japan, 2003–2019. Average dietary scores by survey year were analysed by linear regression analysis. All dietary pattern trends were significant (*P* < 0·001). Dietary pattern 1: ‘Japanese style’ pattern, Dietary pattern 2: ‘bread and dairy’ pattern, Dietary pattern 3: ‘meat and oil’ pattern, and Dietary pattern 4: ‘noodles’ pattern

Figure 1 displays 17-year trends of dietary patterns for each sex. In both sexes, while the ‘Japanese style’ pattern’s scores decreased over the study period, those of the ‘bread and dairy’ pattern increased. In the other two patterns exclusively identified in men, the ‘meat and oil’ pattern’s scores also exhibited an increasing trend, whereas the ‘noodles’ scores remained relatively stable. These trends in the identified dietary patterns were consistent with food intake trends (Table 2).

**Table 2:** The mean nutrient intakes, NRF9·3 scores and PFC ratios for Japanese adults in the highest quartile of each dietary pattern scores for 67·066 women and 55 133 men of the NHNSJ 2003–2019 survey by gender. 16 767 women and 13 784 men were extracted as subjects in the highest quartile of each dietary pattern

		Women				Men							
		Japanese style pattern		Bread and dairy pattern		Japanese style pattern		Bread and dairy pattern		Meat and oil pattern		Noodles pattern	
Nutrients		Mean	sd	Mean	sd	Mean	sd	Mean	sd	Mean	sd	Mean	sd
Energy	kcal	2046·0	432·6	1934·1	458·4	2491·4	571·1	2291·3	569·3	2547·5	579·3	2148·6	596·5
Total protein	g	81·9	20·3	72·1	20·3	95·8	24·8	84·0	24·3	89·9	24·2	78·9	25·2
Animal protein	g	43·1	17·9	38·4	16·6	51·1	22·2	44·0	19·8	51·7	20·1	39·6	20·2
Plant protein	g	38·8	10·1	33·7	10·2	44·7	11·9	40·0	12·2	38·2	11·9	39·3	12·0
Total fat	g	58·7	23·1	66·8	23·0	64·0	26·4	67·7	27·0	83·3	25·8	59·0	26·5
Animal fat	g	28·6	15·2	32·6	16·0	33·1	18·6	33·6	18·3	43·1	19·9	29·8	18·4
Plant fat	g	30·0	15·7	34·2	16·3	30·9	16·4	34·1	18·0	40·2	18·0	29·1	16·3
Carbohydrate	g	292·1	69·4	253·0	70·5	346·7	91·5	318·0	86·6	324·9	93·6	284·6	84·5
Na	mg	5128·1	1664·6	4102·5	1497·4	5959·1	1905·4	4692·0	1761·0	5015·6	1828·0	5229·7	1871·4
Potassium	mg	3304·3	850·5	2586·5	943·5	3386·1	929·3	3015·8	1034·3	2684·8	938·2	2470·7	956·9
Ca	mg	719·4	285·0	616·3	289·2	693·8	288·4	704·7	317·5	537·9	269·9	531·9	272·9
Mg	mg	337·8	87·6	263·3	92·6	369·3	96·5	314·0	104·7	288·5	97·1	284·3	102·9
Phosphorus	mg	1224·9	302·1	1067·6	320·4	1369·2	344·3	1239·1	372·3	1218·9	347·5	1103·6	365·2
Fe	mg	10·8	6·0	8·3	4·7	11·5	3·4	9·5	3·6	9·3	3·3	8·7	3·5
Vitamin A	μg	831·2	776·9	656·2	671·9	833·3	1017·9	758·5	1002·8	691·0	940·6	589·4	837·9
Vitamin D	μg	11·1	10·2	6·5	7·7	12·8	11·8	9·0	9·6	7·3	8·5	7·6	9·0
Vitamin E	mg	11·2	20·6	9·8	18·9	10·5	16·5	10·1	18·0	9·6	10·5	8·1	14·4
Vitamin K	μg	365·2	225·3	251·4	185·7	385·1	238·9	282·2	207·5	291·7	199·0	255·3	197·9
Vitamin B_1_	mg	1·6	5·8	1·4	4·8	1·7	5·4	1·6	5·5	1·4	3·3	1·4	4·4
Vitamin B_2_	mg	1·8	9·4	1·5	3·2	1·8	9·8	1·8	9·8	1·5	1·8	1·4	2·0
Niacin	mg	19·2	8·4	16·9	8·3	23·0	10·6	19·8	9·9	21·5	10·5	19·4	9·7
Vitamin B_6_	mg	2·1	6·1	1·7	5·4	2·2	5·1	1·9	5·0	1·7	3·1	1·6	4·3
Vitamin B_12_	μg	8·7	7·8	5·6	5·9	10·6	9·8	7·9	8·1	6·5	7·2	7·2	8·1
Folic acid	μg	439·8	148·7	333·0	153·0	446·8	171·0	386·1	176·7	342·1	165·8	317·0	163·9
Pantothenic acid	mg	6·8	1·8	5·9	1·9	7·4	2·1	6·8	2·2	6·9	2·0	5·9	2·1
Vitamin C	mg	187·4	169·6	140·1	152·4	157·6	127·8	154·8	135·6	112·7	124·0	102·6	114·6
SFA	g	15·1	7·2	19·1	7·7	15·8	7·8	19·2	8·9	21·8	8·7	15·7	8·4
MFA	g	19·4	9·1	23·2	9·4	21·5	10·5	22·9	10·7	30·7	10·5	20·2	10·4
PFA	g	14·0	6·0	13·4	6·0	15·7	6·6	14·2	6·5	17·9	6·5	13·3	6·2
Cholesterol	mg	351·2	186·5	346·6	183·7	407·8	221·2	360·2	198·8	468·9	217·3	324·5	196·6
Fibre	g	22·0	7·3	17·1	7·2	22·0	7·6	19·4	7·9	17·0	7·3	16·7	7·3
Salt	g	13·0	4·2	10·4	3·8	15·1	4·8	11·9	4·5	12·7	4·6	13·3	4·8
Sugar	g	11·9	12·2	8·9	11·4	10·8	12·2	10·8	13·3	8·0	10·9	6·3	8·3
Nutrition evaluation													
NRF9·3 scores		716·0	87·1	636·9	121·7	670·5	103·3	661·2	118·1	566·2	120·8	574·7	133·6
PFC ratio													
Protein	%	16·2	3·0	15·0	2·8	15·6	3·1	14·7	2·8	14·3	2·7	14·9	3·1
Fat	%	25·4	7·0	31·0	6·9	22·9	6·8	26·2	6·9	29·7	6·5	24·5	7·4
Carbohydrate	%	58·4	8·0	54·0	7·6	61·5	8·0	59·1	7·7	56·0	7·4	60·6	8·4

PFC ratio, a ratio of energy from protein, fat, and carbohydrates; NRF9.3 scores, Nutrient-Rich Food Index 9.3. Tukey–Kramer tests were performed for nutrient intake in the dietary patterns. The results of the tests were shown in the supplementary material.

### Nutrient intakes of different dietary pattern

Except for a few nutrients, most of the mean values of nutrient intakes, NRF9·3 scores and PFC ratio within the highest quartiles (i.e. those with the highest principal component scores) between different dietary patterns were significantly different (Table 2). The details on *P* values of mean nutrient intakes, NRF9·3 scores and PFC ratios between each dietary pattern are presented in see online supplementary material, Supplemental Tables S3a and S3b.

In both sexes, high agreement with the ‘Japanese style’ pattern had higher total carbohydrates, most micronutrients and salt but lower total fat, SFA and MUFA intake. In contrast, high scores on the ‘bread and dairy’ pattern had lower Na intake but higher K, Ca, Mg, P, Fe, vitamin A, vitamin D, vitamin C and SFA. Men with high scores for the ‘meat and oil’ pattern tended to be high in total fat, SFA, MUFA, PUFA and cholesterol but low in total protein and fibre intake. High agreement with the ‘noodles’ pattern had lower intakes of total protein and most micronutrients but higher salt intake. This pattern also tended to be low in total fat, SFA, MUFA, PUFA and cholesterol intake.

The nutrition assessment result showed that the highest quartiles of the ‘Japanese style’ pattern had higher NRF9·3 scores, with those of women being the highest (716·0). Contrarily, the NRF9·3 score was lower for those of the ‘meat and oil’ pattern, with men’s being the lowest (566·2). NRF9·3 scores for the ‘bread and dairy’ (636·9 in women, 661·2 in men), and ‘noodles’ (574·3) patterns were between the two former patterns.

The PFC results showed that the high quartile of the ‘Japanese style’ pattern (16·2:25·4 for women and 15·4:23·16 for men) was highest than the other dietary patterns. Among men, the ‘meat and oil’ pattern was the lowest (14·1:29·4), while the ‘bread and dairy’ (14·7:26·6) and ‘noodles’ (14·7:24·7) patterns were intermediate between the two patterns.

## Discussion

### Dietary patterns and the trends

We identified two common dietary patterns in Japanese women and men: the ‘Japanese style’ and the ‘bread and dairy’ pattern. Additionally, two other patterns: the ‘meat and oil’ and ‘noodles’ were pronounced only in men.

The ‘Japanese style’ pattern, loaded on sea products and vegetables, was similar to the ‘plant food and fish’^(10)^, ‘Japanese’^(23)^, ‘vegetable’^(24)^ or ‘fish and vegetable’^(12)^ pattern, in previous Japanese studies. The ‘Japanese style’ pattern could be considered the diet recommended by the Ministry of Agriculture, Forestry and Fisheries around the 1975s in Japan^(25,26)^. The ‘Japanese style’ pattern is a well-balanced diet consisting of a staple meal of rice, sea products and side dishes of vegetables, mushrooms, seaweed and tubers and roots^(25,26)^. However, in women, the ‘Japanese style’ pattern showed a rice intake that was below the threshold of loadings. This may indicate that the ‘Japanese style’ pattern after 2000 no longer involves consuming much rice. The results of this study reflect the continued decline in rice consumption, particularly since 2000, showing that rice consumption is low^(27)^. Accordingly, this pattern also had a high loading of Japanese seasonings, which are the main contributors to Na intake in the Japanese diet^(28)^. A Japanese dietary pattern generally has aspects that are similar to healthy dietary patterns among Western populations, characterised by a high load of vegetables and fruits^(29)^. The ‘bread and dairy’ pattern was comparable to what has been previously referred to as the ‘Westernised breakfast’ and ‘bread and confectioneries’ patterns^(10,30)^. The ‘meat and oil’ pattern is comparable to the patterns reported previously among Japanese populations, commonly loaded on meat, animal products, refined grains, soft drinks and coffee^(10,12,31)^. It is worth noting that there was sex difference in food intake characteristics of the ‘meat and oil’ pattern in which men consuming more varied animal foods and saturated fat-rich foods than their women counterparts (Table 2). In this study, the ‘meat and oil’ pattern was extracted only for men^(10,30)^.

### Dietary pattern transition

The ‘meat and oil’ and ‘bread and dairy’ patterns represent Westernised diet as both patterns had a high intake of key foods similar to those in the Western diet (e.g. meat, bread and dairy) but low of those in Japanese diet (e.g. rice, miso soup, soya product and fish). Results of a 17-year trend analysis (2003–2019) revealed that the scores of the ‘Japanese style’ pattern decreased while the two Westernised patterns increased and the ‘noodles’ pattern relatively remained steady over time. This supports the previous NHNSJ study findings on the continuous Westernisation of diets in Japan identified from 2003 to 2015^(10)^ and, more broadly, from 1960 to 2005 by food balance sheets^(9)^. This change in dietary patterns is consistent with a decrease in fish intake and an increase in meat intake observed over the past two decades^(7,32,33)^ and reflected in the food group intakes of those in the highest quartile of each pattern in the present study (Table 2). The decreasing trend of the ‘Japanese style’ may also explain the recent national report on the declining consumption of salt^(19,33)^, one of the main contributors to this pattern.

Changes in a nation’s diet are driven by many factors, such as economic development, advances in food production, universal dietary preferences, behavioural changes or cultural preferences and the dynamic linkages among these factors^(34)^. In Japan, the crucial combination that led to a dietary transition has been the rise in per capita disposable income from decades of strong economic growth, which allowed consumers to adopt different diets, and the upsurge of highly productive agricultural and animal husbandry practices, which made it possible to produce more than enough food (e.g. meat, milk and eggs) to meet the growing demand^(2)^. Increases in food energy intake have been linked to significant changes in the shares of key nutrients in most national dietary transitions. According to China’s data, in the previous two decades, both urban and rural people have decreased their consumption of cereals and coarse grains while simultaneously increasing their consumption of animal foods, indicating a preference shift from carbohydrates to fat and protein^(35,36)^. The increasing trend of Westernised patterns in our study provides additional evidence for this shift.

### Nutrient intakes of different dietary patterns

In general, contrasting three other patterns, the ‘Japanese style’ had more features conducive to better health, as reflected by its higher NRF9·3 score compared with other identified patterns (716·0 in women, 670·5 in men) (Table 2). Mean nutrient intakes of those in the highest quartile of this pattern are consistent with dietary guidelines^(37)^, except for Ca in men. High intake of two limiting nutrients considered in NRF9·3 assessment (i.e. Na and sugar) did not negate the effect of all beneficial nutrients (i.e. potassium, Ca, Mg, Fe, vitamin A, vitamin C, vitamin D and fibre), which were the highest across the identified patterns. Therefore, a decreasing trend in the ‘Japanese style’ pattern suggests a potential decline in the intake of those nutrients over time. Based on the NRF9·3 score, the ‘bread and dairy’ pattern had a better nutritional profile (636·9 in women, 661·2 in men) than the ‘meat and oil’ (566·2) – resulting from higher intake of the beneficial nutrients and lower Na intake. In women and men, the ‘bread and dairy’ pattern had low Na intake, and similar patterns were also associated with lower Na intake in previous Japanese studies^(12,30)^. The ‘meat and oil’ pattern had a low NRF9·3 score (566·2), largely owing to a lower intake of the beneficial nutrients, while higher SFA intake, particularly in men. The ‘noodles’ pattern’s nutritional profile was also less favourable (574·3), mainly due to lower protein, fibre and SFA, while higher Na intake. Our food intake data show that the high Na intake in this pattern may be because of the high consumption of salt-based seasonings – an important component in noodle soup. Accordingly, Fujiwara *et al.* (2016)^(12)^ reported that a noodle pattern contributed to higher Na intake among Japanese adults, attributable to the high intake of noodle soup.

Although the ‘Japanese style’ pattern had more health-promoting characteristics, a high salt intake was also observed among those in the highest quartile of this pattern. Similarly, Fujiwara *et al.* (2016)^(12)^ found that a dietary pattern comparable to the ‘Japanese style’ pattern was associated with high salt intake in Japan. High salt intake in the ‘Japanese style’ pattern is likely attributed to salty foods, such as fish products, pickled vegetables, and salt-containing seasonings, including Japanese seasonings^(33,37)^. Widely used Japanese seasonings such as miso and nukazuke (ingredients to make vegetable pickles) contain high salt concentrations^(28)^. Salt intakes across all patterns are higher than Japan’s dietary reference intakes of 6·5–7·5 g/d^(37)^, indicating that the ongoing efforts for salt reduction must be addressed independently of dietary patterns.

The PFC ratio of the ‘Japanese style’ pattern in the present study is comparable to the 1975–2000 Japanese PFC ratio^(38)^, with the highest protein-to-fat ratio compared with other identified patterns. Our findings that the ‘meat and oil’ and ‘bread and dairy’ patterns – the Westernised pattern – increased over the study period implied an increasing trend in fat intake. However, in general, the Westernised patterns in Japan had lower fat and protein proportions in the PFC ratios than in Western countries, such as France and the USA^(38)^.

Several limitations of this study need to be mentioned. First, despite aiming for nationally representative data, the NHNSJ only has about 50 % household response rates. In addition, data on the characteristics of households that declined to participate and individual-level response rates were unavailable^(39)^. Consequently, while the datasets should accurately represent Japanese dietary patterns at the population level, selection bias cannot be ruled out. Second, self-reported dietary record data were used to determine dietary intake, which may be subject to individual interpretation bias. The identified dietary patterns may not represent the participants’ usual patterns, as they were based on a one-day weighted household dietary record. Moreover, given that the survey was performed within a single month (November), the possibility of seasonal variation was also not considered. However, the assessment method can provide accurate population averages^(10)^. Third, principal component analysis is subject to several limitations stemming from arbitrary analytical decisions that might have affected both the results and their interpretation. These include food group numbers and categorisation, extracted components, rotation technique, component labelling and interpretation. Thus, we applied measures in the objective decision-making method, including eigenvalues, and interpretability in identifying dietary patterns^(12)^. Fourth, while the trends in energy and nutrient intakes observed in this study were generally consistent, the possibility of a data continuity issue cannot be excluded because we did not recalculate the NHNSJ survey result based on the updated STFC. An analysis of multiyear NHNSJ data may lead to a continuity issue due to the STFC revisions, thus changing the survey results^(33,40)^. Although the continuity of the calculation using different versions of the STFC in Japan has been poorly examined^(20)^, some studies were against recalculating previous dietary survey data as there were no abrupt changes in dietary intake trends. For instance, a minor change in mean energy, protein and fat intake (approximately <2 %) was reported with the revision from the fourth to the fifth version^(20,33)^. Saito *et al.* (2018)^(33)^ also confirmed that other revisions did not include significant changes in the calculation method or nutrient analysis. Nonetheless, our results suggest that caution should be warranted in interpreting the population estimates. Additionally, due to a data paucity, food intake variations across regions as well as urban and rural areas^(21)^ could not be considered.

## Conclusion

The present study identified two major dietary patterns among Japanese adults: the ‘Japanese style’ and ‘bread and dairy’ patterns. Additionally, the ‘meat and oil’ and ‘noodles’ patterns were only pronounced in men. The nutritional assessment showed that the ’Japanese style’ is the most favorable but its scores decreased over time suggesting the continuous Westernisation of the Japanese diet. The decreasing trend of dietary patterns with the most preferable nutrient profile might indicate a potential decrease in beneficial nutrient intakes and, thus, a deterioration of the nutritional status of the Japanese population. The findings can be used to examine the association between diet and health outcomes, to develop food-based dietary guidelines and to call for continuous monitoring and evaluation of dietary patterns for opportunities to improve the population’s diet and health.

## Supporting information

Sakai et al. supplementary material 1 Sakai et al. supplementary material

Sakai et al. supplementary material 2 Sakai et al. supplementary material

Sakai et al. supplementary material 3 Sakai et al. supplementary material

## Supplementary material

For supplementary material accompanying this paper visit https://doi.org/10.1017/S1368980024002027
