# Supplementary material for: Dietary pattern transition and its nutrient intakes and diet quality among Japanese population: results from the 2003–2019 National Survey
Source: Public Health Nutr. 2024 Oct 21;27(1):e231. doi: 10.1017/S1368980024002027 (PMC11645119; doi:10.1017/S1368980024002027)
Supplement: Sakai et al. supplementary material 1 — Sakai et al. supplementary material [file S1368980024002027sup001.docx]

Table S1. Categorization of the food items based on functional food ingredients.

| **Food item** | **Food groups categories** |
| --- | --- |
| Rice | Rice |
| Processed rice products | Rice |
| Wheat flour | Bread |
| Breads (except sweet bread) | Bread |
| Sweet bread | Confectionaries |
| Udon noodles & Chinese noodles | Noodles |
| Instant Chinese noodles | Noodles |
| Pasta | Noodles |
| Other processed wheat products | Bread |
| Soba & processed foods | Noodles |
| Corn & processed products | Other grains |
| Other grains | Other grains |
| Sweet potatoes & processed products | Potatoes |
| Potatoes & processed products | Potatoes |
| Other tubers & processed products | Potatoes |
| Starch & processed products | Potatoes |
| Sugar & sweeteners | Sugar |
| Soybeans (whole) & processed products | Pulses |
| Tofu | Pulses |
| Fried tofu | Pulses |
| Natto | Pulses |
| Other processed soybean products | Pulses |
| Other beans & processed foods | Pulses |
| Nuts | Nuts |
| Tomato | Green and yellow vegetables |
| Carrot | Green and yellow vegetables |
| Spinach | Green and yellow vegetables |
| Green pepper | Green and yellow vegetables |
| Other green & yellow vegetables | Green and yellow vegetables |
| Cabbage | Other vegetables |
| Cucumber | Other vegetables |
| Daikon radish | Other vegetables |
| Onions | Other vegetables |
| Chinese cabbage | Other vegetables |
| Other hypochromic vegetable | Other vegetables |
| Vegetable juice | Vegetable and fruit juice |
| Pickled leaves | Pickled vegetables |
| Pickled radish & other pickled vegetables | Pickled vegetables |
| Strawberry | Fruits |
| Citrus fruits | Fruits |
| Banana | Fruits |
| Apple | Fruits |
| Other fruits | Fruits |
| Jam | Sugar |
| Fruit juice & fruit juice beverages | Vegetable and fruit juice |
| Mushrooms | Mushrooms |
| Seaweeds | Seaweeds |
| Horse mackerel & sardines | Fish and seafood |
| Salmon & salmon trout | Fish and seafood |
| Snapper & flatfish | Fish and seafood |
| Tuna & swordfish | Fish and seafood |
| Other raw fish | Fish and seafood |
| Shellfish | Fish and seafood |
| Squid & octopus | Fish and seafood |
| Shrimp & crabs | Fish and seafood |
| Seafood (salted, dried, dried) | Fish and seafood |
| Seafood (canned) | Fish and seafood |
| Seafood (tsukudani) | Fish and seafood |
| Seafood (fish paste products) | Fish and seafood |
| Fish meat ham & sausages | Fish and seafood |
| Beef | Red meat |
| Pork | Red meat |
| Ham & sausages | Processed meat |
| Other meat | Red meat |
| Chicken | Chicken |
| Other poultry | Chicken |
| Other meat & processed foods | Processed meat |
| Eggs | Eggs |
| Milk | Dairy products |
| Cheese | Dairy products |
| Fermented milk & lactic acid beverages | Dairy products |
| Other dairy products | Dairy products |
| Other dairy products | Dairy products |
| Butter | Animal fat |
| Margarine | Vegetable oil |
| Vegetable oils | Vegetable oil |
| Animal oils | Animal fat |
| Other fats | Vegetable oil |
| Japanese cakes | Confectionaries |
| Cakes & pastries | Confectionaries |
| Biscuits | Confectionaries |
| Candies | Confectionaries |
| Other confectioneries | Confectionaries |
| Sake | Alcoholic drinks |
| Beer | Alcoholic drinks |
| Western liquor & others | Alcoholic drinks |
| Japanese tea | Tea |
| Coffee & cocoa | Coffee |
| Sugar-sweetened beverages and other beverages | Soft drinks |
| Sauces | Salt-based seasonings |
| Soy sauce | Japanese seasoning |
| Salt | Salt-based seasonings |
| Mayonnaise | Eggs |
| Miso | Japanese seasoning |
