# Supplementary material for: Dietary pattern transition and its nutrient intakes and diet quality among Japanese population: results from the 2003–2019 National Survey
Source: Public Health Nutr. 2024 Oct 21;27(1):e231. doi: 10.1017/S1368980024002027 (PMC11645119; doi:10.1017/S1368980024002027)
Supplement: Sakai et al. supplementary material 2 — Sakai et al. supplementary material [file S1368980024002027sup002.docx]

Table S2. Sample characteristics for the 122,199 adults (aged over 18 years) of the National Health and Nutrition Survey, Japan 2003-2019.

| Year | | 2003 | 2004 | 2005 | 2006 | 2007 | 2008 | 2009 | 2010 | 2011 | 2012 | 2013 | 2014 | 2015 | 2016 | 2017 | 2018 | 2019 | *p-*value |
| --- | --- | --- | --- | --- | --- | --- | --- | --- | --- | --- | --- | --- | --- | --- | --- | --- | --- | --- | --- |
| Sample size | | 7413 | 5944 | 5751 | 6336 | 6166 | 6411 | 6269 | 5744 | 5446 | 20297 | 5593 | 5504 | 5029 | 16966 | 4633 | 4778 | 3919 |  |
| Gender | |  |  |  |  |  |  |  |  |  |  |  |  |  |  |  |  |  | 1.000 |
|  | Men, n (%) | 3311 (44.6) | 2658 (44.7) | 2588 (45) | 2845 (44.9) | 2808 (45.5) | 2893 (45.1) | 2817 (44.9) | 2568 (44.7) | 2459 (45.1) | 9028 (44.4) | 2578 (46) | 2534 (46) | 2265 (45) | 7624 (44.9) | 2142 (46.2) | 2233 (46.7) | 1786 (45.5) |  |
|  | Women, n (%) | 4206 (55.4) | 3287 (55.3) | 3163 (55) | 3491 (55.1) | 3358 (54.5) | 3518 (54.9) | 3452 (55.1) | 3176 (55.3) | 2987 (54.9) | 11269 (55.6) | 3015 (54) | 2970 (54) | 2764 (55) | 9342 (55.1) | 2491 (53.8) | 2545 (53.3) | 2133 (54.5) |  |
| Age | |  |  |  |  |  |  |  |  |  |  |  |  |  |  |  |  |  | 0.000 |
|  | 18-39 years | 1954 | 1583 | 1375 | 1627 | 1502 | 1386 | 1416 | 1254 | 1210 | 4072 | 1156 | 1005 | 870 | 2919 | 772 | 834 | 603 |  |
|  | 40-59 years | 2521 | 1995 | 1898 | 2143 | 2041 | 1994 | 2022 | 1787 | 1632 | 6029 | 1623 | 1627 | 1587 | 5012 | 1423 | 1510 | 1182 |  |
|  | Over 60 years | 2938 | 2366 | 2478 | 2566 | 2623 | 3031 | 2831 | 2703 | 2604 | 10196 | 2814 | 2872 | 2572 | 9035 | 2438 | 2434 | 2134 |  |
| BMI (kg/m2) | | 22.9±3.5 | 22.9±3.5 | 23.0±3.5 | 23.0±3.4 | 22.9±3.6 | 22.9±3.5 | 22.9±3.6 | 22.9±3.5 | 22.9±3.5 | 23.0±3.5 | 22.9±3.6 | 22.9±3.5 | 22.9±3.6 | 23.1±3.6 | 23.1±3.6 | 23.1±3.6 | 23.1±3.7 | 0.000 |
| Weight status | |  |  |  |  |  |  |  |  |  |  |  |  |  |  |  |  |  | 0.5289 |
|  | Underweight, n (%) | 564 (7.6) | 437 (7.3) | 395 (6.8) | 436 (6.8) | 436 (7) | 490 (7.6) | 486 (7.7) | 446 (7.7) | 423 (7.7) | 1520 (7.4) | 484 (8.6) | 421 (7.6) | 375 (7.4) | 1298 (7.6) | 334 (7.2) | 361 (7.5) | 302 (7.7) |  |
|  | Normal weight, n (%) | 4984 (67.1) | 4044 (68) | 3893 (67.6) | 4303 (67.9) | 4303 (69.7) | 4363 (68) | 4190 (66.8) | 3817 (66.4) | 3640 (66.8) | 13645 (67.2) | 3742 (66.9) | 3706 (67.3) | 3414 (67.8) | 11189 (65.9) | 3081 (66.5) | 3123 (65.3) | 2542 (64.8) |  |
|  | Overweight, n (%) | 1865 (25.3) | 1463 (24.7) | 1463 (25.6) | 1597 (25.3) | 1597 (23.3) | 1558 (24.4) | 1593 (25.5) | 1481 (25.9) | 1383 (25.5) | 5132 (25.4) | 1367 (24.5) | 1377 (25.1) | 1240 (24.8) | 4479 (26.5) | 1218 (26.3) | 1294 (27.2) | 1075 (27.5) |  |

BMI was determined by dividing weight (kg) by height squared (m^2^). Weight status was defined based on BMI: underweight (<18.5 kg/m^2^), normal weight (≥18.5 to <25 kg/m^2^), and overweight and obese (≥25 kg/m^2^).
