# Supplementary material for: Dietary pattern transition and its nutrient intakes and diet quality among Japanese population: results from the 2003–2019 National Survey
Source: Public Health Nutr. 2024 Oct 21;27(1):e231. doi: 10.1017/S1368980024002027 (PMC11645119; doi:10.1017/S1368980024002027)
Supplement: Sakai et al. supplementary material 3 — Sakai et al. supplementary material [file S1368980024002027sup003.docx]

Table S3a. The mean nutrient intakes, NRF9.3 scores, and PFC ratios for Japanese adults in the highest quartile of each dietary pattern scores for 67.066 women and 55,133 men of the National Health and Nutrition Survey, Japan 2003-2019 survey and *p*-value by food groups (women)

| Nutrients | | |  | Women | | | | *p*-value |
| --- | --- | --- | --- | --- | --- | --- | --- | --- |
|  |  |  |  | Japanese style pattern | | Bread and dairy pattern | |  |
|  |  |  |  | Mean | SD | Mean | SD |  |
| Energy | | kcal | | 2046.0 | 432.6 | 1934.1 | 458.4 | * |
| Total protein | | g |  | 81.9 | 20.3 | 72.1 | 20.3 | * |
|  | Animal protein | g |  | 43.1 | 17.9 | 38.4 | 16.6 | * |
|  | Plant protein | g |  | 38.8 | 10.1 | 33.7 | 10.2 | * |
| Total fat | | g |  | 58.7 | 23.1 | 66.8 | 23.0 | * |
|  | Animal fat | g |  | 28.6 | 15.2 | 32.6 | 16.0 | * |
|  | Plant fat | g |  | 30.0 | 15.7 | 34.2 | 16.3 | * |
| Carbohydrate | | g |  | 292.1 | 69.4 | 253.0 | 70.5 | * |
| Sodium | | mg |  | 5128.1 | 1664.6 | 4102.5 | 1497.4 | * |
| Potassium | | mg |  | 3304.3 | 850.5 | 2586.5 | 943.5 | * |
| Calcium | | mg |  | 719.4 | 285.0 | 616.3 | 289.2 | * |
| Magnesium | | mg |  | 337.8 | 87.6 | 263.3 | 92.6 | * |
| Phosphorus | | mg |  | 1224.9 | 302.1 | 1067.6 | 320.4 | * |
| Iron | | mg |  | 10.8 | 6.0 | 8.3 | 4.7 | * |
| Vitamin A | | μg |  | 831.2 | 776.9 | 656.2 | 671.9 | * |
| Vitamin D | | μg |  | 11.1 | 10.2 | 6.5 | 7.7 | * |
| Vitamin E | | mg |  | 11.2 | 20.6 | 9.8 | 18.9 | * |
| Vitamin K | | μg |  | 365.2 | 225.3 | 251.4 | 185.7 | * |
| Vitamin B_1_ | | mg |  | 1.6 | 5.8 | 1.4 | 4.8 | * |
| Vitamin B_2_ | | mg |  | 1.8 | 9.4 | 1.5 | 3.2 | * |
| Niacin | | mg |  | 19.2 | 8.4 | 16.9 | 8.3 | * |
| Vitamin B_6_ | | mg |  | 2.1 | 6.1 | 1.7 | 5.4 | * |
| Vitamin B_12_ | | μg |  | 8.7 | 7.8 | 5.6 | 5.9 | * |
| Folic acid | | μg |  | 439.8 | 148.7 | 333.0 | 153.0 | * |
| Pantothenic acid | | mg |  | 6.8 | 1.8 | 5.9 | 1.9 | * |
| Vitamin C | | mg |  | 187.4 | 169.6 | 140.1 | 152.4 | * |
| SFAs | | g |  | 15.1 | 7.2 | 19.1 | 7.7 | * |
| MFAs | | g |  | 19.4 | 9.1 | 23.2 | 9.4 | * |
| PFAs | | g |  | 14.0 | 6.0 | 13.4 | 6.0 | * |
| Cholesterol | | mg |  | 351.2 | 186.5 | 346.6 | 183.7 |  |
| Fiber | | g |  | 22.0 | 7.3 | 17.1 | 7.2 | * |
| Salt | | g |  | 13.0 | 4.2 | 10.4 | 3.8 | * |
| Sugar | | g |  | 11.9 | 12.2 | 8.9 | 11.4 | * |
| Nutrition evaluation | |  |  |  |  |  |  |  |
|  | NRF9.3 scores |  |  | 716.0 | 87.1 | 636.9 | 121.7 | * |
| PFC ratio | |  |  |  |  |  |  |  |
|  | Protein ratio | % |  | 16.2 | 3.0 | 15.0 | 2.8 | * |
|  | Fat ratio | % |  | 25.4 | 7.0 | 31.0 | 6.9 | * |
|  | Carbohydrate ratio | % |  | 58.4 | 8.0 | 54.0 | 7.6 | * |

Table S3b. The mean nutrient intakes, NRF9.3 scores, and PFC ratios for Japanese adults in the highest quartile of each dietary pattern scores for 67.066 women and 55,133 men of the National Health and Nutrition Survey, Japan 2003-2019 survey and *p*-value by food groups (men)

| Nutrients | | |  | Men | | | | *p*-value ^(1)^ | | | | | |
| --- | --- | --- | --- | --- | --- | --- | --- | --- | --- | --- | --- | --- | --- |
|  |  |  |  | Japanese style pattern | Bread and dairy pattern | Meat and oil pattern | Noodles pattern | Japanese style pattern & Bread and dairy pattern | Japanese style pattern & Meat and oil pattern | Japanese style pattern & Noodles pattern | Bread and dairy pattern & Meat and oil pattern | Bread and dairy pattern & Noodles pattern | Meat and oil pattern & Noodles pattern |
|  |  |  |  | Mean | Mean | Mean | Mean |  |  |  |  |  |  |
| Energy | | kcal | | 2491.4 | 2291.3 | 2547.5 | 2148.6 | * | * | * | * | * | * |
| Total protein | | g |  | 95.8 | 84.0 | 89.9 | 78.9 | * | * | * | * | * | * |
|  | Animal protein | g |  | 51.1 | 44.0 | 51.7 | 39.6 | * |  | * | * | * | * |
|  | Plant protein | g |  | 44.7 | 40.0 | 38.2 | 39.3 | * | * | * | * | * | * |
| Total fat | | g |  | 64.0 | 67.7 | 83.3 | 59.0 | * | * | * | * | * | * |
|  | Animal fat | g |  | 33.1 | 33.6 | 43.1 | 29.8 |  | * | * | * | * | * |
|  | Plant fat | g |  | 30.9 | 34.1 | 40.2 | 29.1 | * | * | * | * | * | * |
| Carbohydrate | | g |  | 346.7 | 318.0 | 324.9 | 284.6 | * | * | * |  | * | * |
| Sodium | | mg |  | 5959.1 | 4692.0 | 5015.6 | 5229.7 | * | * | * | * | * | * |
| Potassium | | mg |  | 3386.1 | 3015.8 | 2684.8 | 2470.7 | * | * | * | * | * | * |
| Calcium | | mg |  | 693.8 | 704.7 | 537.9 | 531.9 |  | * | * | * | * |  |
| Magnesium | | mg |  | 369.3 | 314.0 | 288.5 | 284.3 | * | * | * | * | * |  |
| Phosphorus | | mg |  | 1369.2 | 1239.1 | 1218.9 | 1103.6 | * | * | * |  | * | * |
| Iron | | mg |  | 11.5 | 9.5 | 9.3 | 8.7 | * | * | * | * | * | * |
| Vitamin A | | μg |  | 833.3 | 758.5 | 691.0 | 589.4 | * | * | * | * | * | * |
| Vitamin D | | μg |  | 12.8 | 9.0 | 7.3 | 7.6 | * | * | * | * | * |  |
| Vitamin E | | mg |  | 10.5 | 10.1 | 9.6 | 8.1 |  | * | * |  | * | * |
| Vitamin K | | μg |  | 385.1 | 282.2 | 291.7 | 255.3 | * | * | * | * | * | * |
| Vitamin B_1_ | | mg |  | 1.7 | 1.6 | 1.4 | 1.4 |  | * | * |  | * |  |
| Vitamin B_2_ | | mg |  | 1.8 | 1.8 | 1.5 | 1.4 |  |  | * |  | * |  |
| Niacin | | mg |  | 23.0 | 19.8 | 21.5 | 19.4 | * | * | * | * |  | * |
| Vitamin B_6_ | | mg |  | 2.2 | 1.9 | 1.7 | 1.6 | * | * | * | * | * |  |
| Vitamin B_12_ | | μg |  | 10.6 | 7.9 | 6.5 | 7.2 | * | * | * | * | * | * |
| Folic acid | | μg |  | 446.8 | 386.1 | 342.1 | 317.0 | * | * | * | * | * | * |
| Pantothenic acid | | mg |  | 7.4 | 6.8 | 6.9 | 5.9 | * | * | * |  | * | * |
| Vitamin C | | mg |  | 157.6 | 154.8 | 112.7 | 102.6 |  | * | * | * | * | * |
| SFAs | | g |  | 15.8 | 19.2 | 21.8 | 15.7 | * | * |  | * | * | * |
| MFAs | | g |  | 21.5 | 22.9 | 30.7 | 20.2 | * | * | * | * | * | * |
| PFAs | | g |  | 15.7 | 14.2 | 17.9 | 13.3 | * | * | * | * | * | * |
| Cholesterol | | mg |  | 407.8 | 360.2 | 468.9 | 324.5 | * | * | * | * | * | * |
| Fiber | | g |  | 22.0 | 19.4 | 17.0 | 16.7 | * | * | * | * | * |  |
| Salt | | g |  | 15.1 | 11.9 | 12.7 | 13.3 | * | * | * | * | * | * |
| Sugar | | g |  | 10.8 | 10.8 | 8.0 | 6.3 |  | * | * | * | * | * |
| Nutrition evaluation | |  |  |  |  |  |  |  |  |  |  |  |  |
|  | NRF9.3 scores |  |  | 670.5 | 661.2 | 574.7 | 574.7 | * | * | * | * | * |  |
| PFC ratio | |  |  |  |  |  |  |  |  |  |  |  |  |
|  | Protein | % |  | 15.6 | 14.7 | 14.3 | 14.9 | * | * | * | * | * | * |
|  | Fat | % |  | 22.9 | 26.2 | 29.7 | 24.5 | * | * | * | * | * | * |
|  | Carbohydrate | % |  | 61.5 | 59.1 | 56.0 | 60.6 | * | * | * | * | * | * |
| (1) *p* for differences among respected components. * Mean intakes were significantly different (p <0.001). Tukey-Kramer tests were performed for nutrient intake in the dietary patterns. | | | | | | | | | | | | | |
